# The Triterpenoid Betulin Protects against the Neuromuscular Effects of *Bothrops jararacussu* Snake Venom *In Vivo*

**DOI:** 10.1155/2015/939523

**Published:** 2015-11-08

**Authors:** Miriéle Cristina Ferraz, Jhones Luiz de Oliveira, Joel Reis de Oliveira Junior, José Carlos Cogo, Márcio Galdino dos Santos, Luiz Madaleno Franco, Pilar Puebla, Helena Onishi Ferraz, Humberto Gomes Ferraz, Marisa Maria Teixeira da Rocha, Stephen Hyslop, Arturo San Feliciano, Yoko Oshima-Franco

**Affiliations:** ^1^Post-Graduate Program in Pharmaceutical Sciences and Pharmacy Course, University of Sorocaba (UNISO), Rodovia Raposo Tavares, Km 92,5, 18023-000 Sorocaba, SP, Brazil; ^2^Serpentarium of the Center for Nature Studies and Institute for Research and Development (IP&D), Vale do Paraíba University (UNIVAP), Avenida Shishima Hifumi 291, 12244-000 São José dos Campos, SP, Brazil; ^3^Post-Graduate Program in Environmental Sciences, Tocantins Federal University, Avenida NS15, ALC NO14, 109 Norte, 77001-090 Palmas, TO, Brazil; ^4^Pharmacy Course, Methodist University of Piracicaba (UNIMEP), Rodovia do Açúcar, Km 156, 13423-170 Piracicaba, SP, Brazil; ^5^Department of Pharmaceutical Chemistry, Salamanca University, Campus “Miguel de Unamuno”, 37007 Salamanca, Spain; ^6^Pharmacy Course, Federal University of São Paulo (UNIFESP), Rua Prof. Artur Riedel 275, 09972-270 Diadema, SP, Brazil; ^7^Department of Pharmacy, Faculty of Pharmaceutical Sciences, University of São Paulo (USP), Avenida Prof. Lineu Prestes 580, 05434-070 São Paulo, SP, Brazil; ^8^Laboratory of Herpetology, Instituto Butantan, Avenida Vital Brasil 1500, 05503-900 São Paulo, SP, Brazil; ^9^Department of Pharmacology, Faculty of Medical Sciences, State University of Campinas (UNICAMP), Rua Tessália Vieira de Camargo 126, Cidade Universitária Zeferino Vaz, 13083-870 Campinas, SP, Brazil

## Abstract

We confirmed the ability of the triterpenoid betulin to protect against neurotoxicity caused by *Bothrops jararacussu* snake venom *in vitro* in mouse isolated phrenic nerve-diaphragm (PND) preparations and examined its capability of *in vivo* protection using the rat external popliteal/sciatic nerve-tibialis anterior (EPSTA) preparation. Venom caused complete, irreversible blockade in PND (40 *μ*g/mL), but only partial blockade (~30%) in EPSTA (3.6 mg/kg, i.m.) after 120 min. In PND, preincubation of venom with commercial bothropic antivenom (CBA) attenuated the venom-induced blockade, and, in EPSTA, CBA given i.v. 15 min after venom also attenuated the blockade (by ~70% in both preparations). Preincubation of venom with betulin (200 *μ*g/mL) markedly attenuated the venom-induced blockade in PND; similarly, a single dose of betulin (20 mg, i.p., 15 min after venom) virtually abolished the venom-induced decrease in contractility. Plasma creatine kinase activity was significantly elevated 120 min after venom injection in the EPSTA but was attenuated by CBA and betulin. These results indicate that betulin given i.p. has a similar efficacy as CBA given i.v. in attenuating the neuromuscular effects of *B. jararacussu* venom *in vivo* and could be a useful complementary measure to antivenom therapy for treating snakebite.

## 1. Introduction

Snakebite is a worldwide health problem that results in at least 20,000 deaths each year [1]. Serum therapy is the primary treatment for systemic envenoming but its efficacy against local effects (pain, edema, hemorrhage, and necrosis) is limited [2]. Consequently, there has been increasing interest in the rapidly expanding field of “green medicine” that includes the study and use of plant products (extracts or isolated components) as complementary or ancillary measures to treat the local effects of snake venoms [3]. Appropriate exploitation of these plants can provide compounds for pharmacological analysis, whilst minimizing the destruction of natural resources, a critical aspect of sustainability [3].

Betulin is an important precursor biomolecule that can be converted to betulinic acid, a C-28 carboxylic derivative that is generally produced by plants in small amounts [4, 5]. However, numerous plants produce large amounts of betulin (Table 1) [6–28]. The clinical effects of betulin, mainly as an anticancer drug, have been pharmacologically less exploited than those of betulinic acid [29, 30]. However, a preliminary pharmacokinetic analysis of betulin found good bioavailability when administered intraperitoneally (i.p.) or subcutaneously (s.c.); there was also no subchronic toxicity in rats (injected i.p.) or dogs (injected s.c.) [9].

An ethnobotanical study previously identified* Dipteryx alata* Vogel as a plant with anti-snake venom properties [31], and Nazato et al. [32] subsequently confirmed this activity for a hydroalcoholic extract of* D. alata* bark. Puebla et al. [15] identified 18 compounds in* D. alata*, including betulin, previously isolated by Coelho Kaplan et al. [14]. Subsequent investigation showed that betulin attenuated the neurotoxicity and myotoxicity of* Crotalus durissus terrificus* (South American rattlesnake) and* Bothrops jararacussu* (jararacuçu) snake venoms* in vitro*, as assessed by twitch-tension recordings (neurotoxicity) and light microscopy (myotoxicity) [33].

*Bothrops jararacussu* venom causes irreversible paralysis* in vitro* [34] and myonecrosis at the bite site [35]. Based on previous findings with* D. alata* and betulin in phrenic nerve-diaphragm (PND) preparations* in vitro* [15, 32, 33], we speculated whether betulin could also attenuate the neuromuscular effects of snake venoms in a nerve-muscle preparation, the rat external popliteal/sciatic nerve-tibialis anterior muscle (EPSTA) preparation* in situ*, particularly when compared to the efficacy of commercial bothropic antivenom (CBA). Since the EPSTA is analyzed* in situ* whilst the rat is kept anesthetized throughout the experiment (120 min), this preparation may provide additional insights that are not immediately obtainable with PND preparations. Previous investigations have shown that the nerves supplying the EPSTA muscle are sensitive to a venom concentration of 40 *μ*g/mL [36, 37], which was also used here. In this study, we therefore confirmed the effects of betulin in mouse PND and examined the neuromuscular alterations (changes in twitch-tension) and myotoxicity (assessed by creatine kinase release) caused by* B. jararacussu* venom in rat EPSTA; we also assessed the effect of betulin on the responses to venom in the latter preparation.

## 2. Material and Methods

### 2.1. Betulin and Its Dispersion

**Figure 1:**
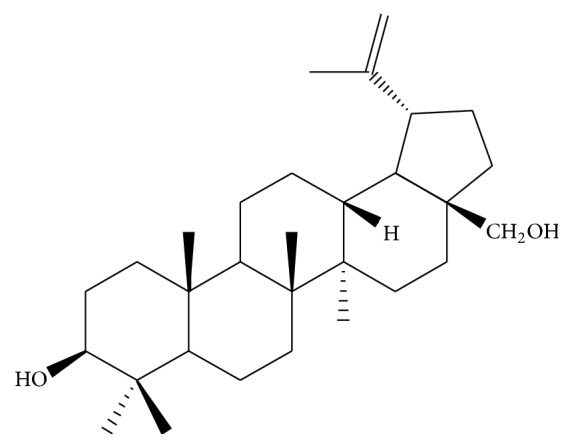
Chemical structure of betulin [15].

The triterpenoid betulin (Figure 1), found in plants such as* D. alata* Vogel [15, 33], was purchased from Sigma Chemical Co. (St. Louis, MO, USA) and used throughout this study.

For* in vitro* experiments, betulin (200 *μ*g/mL) was dispersed in a maximum of 15 *μ*L of polyethylene glycol 400 (PEG 400) prior to addition to the organ bath. The final concentration of PEG 400 (0.3%) in the organ bath did not change the basal responses of the preparation [38, 39]. For* in vivo* experiments, betulin (1–20 mg) was added to PEG 400 (15–300 *μ*L). The dispersed betulin was placed in a glass beaker and the final volume was adjusted to 1 mL with sterile saline followed by gentle stirring. The osmotic pressure of each betulin solution was measured (in duplicate) using an osmometer (Fiske Associates, Norwood, MA, USA) in order to define the route of administration (intravenously (i.v.) or i.p.) (Table 2). PEG is on the Food and Drug Administration's Generally-Recognized-As-Safe (GRAS) compound list for internal consumption [38, 40–42].

### 2.2. Venom and Antivenom

*Bothrops jararacussu* venom was collected manually from two adult specimens in the Serpentarium of the Center for Nature Studies at UNIVAP. The snakes were housed in open-air, concrete-walled pens and maintained under Environmental license SMA 15.380/2012 (São Paulo state environmental agency); they were fed Swiss white mice every two weeks. The venom was certified by Dr. José Carlos Cogo (UNIVAP), lyophilized, and stored at −20°C until used. Commercial bothropic antivenom (lot 091259/C, expiry date for human use: October 2011) produced by the Instituto Butantan (São Paulo, SP, Brazil) against a pool of* Bothrops* venoms (*B. alternatus*,* B. jararaca*,* B. jararacussu*,* B. moojeni*, and* B. neuwiedi*) [43] was kindly donated by the Escritório Regional de Saúde in Piracicaba, SP, Brazil.

### 2.3. Animals

Male Swiss white mice (26–32 g) and male Wistar rats (300–400 g) were purchased from Anilab (Animais de Laboratório, Paulínia, SP, Brazil). The animals were housed at 25 ± 3°C on a 12 h light/dark cycle and had access to food and water* ad libitum*. This project was approved by the institutional Committee for Ethics in Animal Use of Vale do Paraiba University (protocol number A013/CEUA/2011), and the experiments were done within the ethical guidelines established by the Brazilian Society for Laboratory Animal Science (SBCAL).

### 2.4. Mouse PND Preparation

PND preparations [44] were obtained from mice anesthetized with halothane (Cristália, Itapira, SP, Brazil) and killed by exsanguination. The diaphragm was removed and mounted under a tension of 5 g/cm in a 5 mL organ bath containing aerated Tyrode solution (control) of the following composition (mM): NaCl, 137; KCl, 2.7; CaCl_2_, 1.8; MgCl_2_, 0.49; NaH_2_PO_4_, 0.42; NaHCO_3_, 11.9; and glucose, 11.1. After equilibration with 95% O_2_/5% CO_2_ (v/v), the pH of this solution was 7.0. The preparations were stimulated indirectly with supramaximal stimuli (4x threshold, 0.06 Hz, 0.2 ms) delivered from a stimulator (model ESF-15D, Ribeirão Preto, SP, Brazil) to the nerve by bipolar electrodes. Isometric twitch-tension was recorded with a force-displacement transducer (cat. 7003, Ugo Basile, Varese, Italy) coupled to a two-channel Gemini flatbed physiograph device (cat. 7070, Ugo Basile) via a basic preamplifier (cat. 7080, Ugo Basile). Changes in twitch-tension were recorded as described by Farrapo et al. [45]. The preparations were allowed to stabilize for at least 20 min prior to application of the test agents (experiments described below).

Control experiments were done in which PND preparations were incubated with Tyrode solution alone (*n* = 4) while the treatment groups included incubation with Tyrode solution containing betulin (200 *μ*g/mL; *n* = 10), CBA (8 *μ*L/mL; *n* = 4), or* B. jararacussu* venom (40 *μ*g/mL; *n* = 4). The venom and betulin concentrations were chosen based on previous work [33, 46], whereas the concentration of CBA was calculated based on the manufacturer's information that 1 mL of antivenom neutralizes 5 mg of reference* Bothrops jararaca* venom. These same concentrations of betulin (*n* = 11) and CBA (*n* = 4) were used to test their neutralizing capacity against venom (40 *μ*g/mL). For this, venom and the compound of interest were preincubated for 30 min at room temperature (~25°C) prior to screening for residual venom activity (neuromuscular blockade and/or myotoxicity) in PND preparations.

### 2.5. Rat EPSTA Preparation

This preparation was mounted essentially as described elsewhere [36, 37, 47]. Briefly, male Wistar rats were anesthetized with sodium pentobarbital (40 mg/kg, i.p.; Syntec do Brasil, Cotia, SP, Brazil) and additional doses were given as required during the experiment. The trachea was cannulated with a plastic endotracheal tube connected to a rodent ventilator (cat. 7025, Ugo Basile) and artificial ventilation was initiated at a flow of 1.2 mL/kg and respiratory rate of 70 inflations per minute. The left hind limb was immobilized and the popliteal/sciatic nerve tendon for insertion of the tibialis anterior muscle was freed and attached to a force-displacement transducer (cat. 7003, Ugo Basile) coupled to the recording device used in the PND experiments. The sciatic nerve in the popliteal space was stimulated indirectly with supramaximal stimuli (4x threshold, 0.5 Hz, 0.2 ms) delivered from a stimulator (model ESF-15D) to produce maximal twitches of the tibialis anterior muscle. The resting tension of the muscle was adjusted to 20 g to give the greatest evoked twitch-tension. The preparation was allowed to stabilize for at least 20 min before initiating the treatments. The experiments were run for 120 min after the addition of test agents.

Control experiments using this preparation included the injection of saline (120 *μ*L, *n* = 4) and PEG 400 intramuscularly (i.m.) in the left hind limb since saline and PEG 400 were the vehicles for venom and betulin administration, respectively. For experimental treatments, rats were injected with (1) venom (3.6 mg/kg, corresponding to 1.08 mg for a 300 g rat [36]) injected i.m. in 120 *μ*L of saline (*n* = 4), (2) CBA (1.8 *μ*L of CBA in 1 mL; this volume of CBA neutralized 9 *μ*g of venom based on the neutralizing capacity indicated in Section 2.4); the i.v. infusion of antivenom (via a penile vein) was initiated 15 min after venom injection and was infused slowly over 30 min (*n* = 4), and (3) betulin (1 mL of a 20 mg/mL solution, referred to hereafter simply as 20 mg) administered i.p. 15 min after venom injection (*n* = 4).

### 2.6. Myotoxicity Assessed by Creatine Kinase (CK) Release

Since the EPSTA preparation did not lend itself to the simultaneous collection of blood samples for CK quantification, we undertook another series of experiments to assess the ability of CBA and betulin to attenuate venom-induced muscle damage (CK release). For this, four additional groups of rats (4 rats/group) were treated using protocols similar to those of the EPSTA experiments, except that the route of CBA administration was different (i.p. instead of i.v.), namely, (1) saline (negative control), (2) venom alone (positive control), (3) venom + betulin i.p. 15 min later, and (4) venom + CBA i.p. 15 min later. Since our aim in these experiments was to compare the efficacies of CBA and betulin in reducing venom-induced CK release and since betulin was already being given i.p., we chose to also inject CBA i.p. rather than i.v. in order to facilitate comparison with the protection by betulin, even though in clinical practice CBA is not generally administered i.p. After 2 h, blood samples (3–5 mL) were collected from anesthetized rats by cardiac puncture into heparinized tubes, centrifuged to obtain plasma, and stored for a maximum of 2 h at 4°C until CK quantification. CK activity (expressed in units/L, U/L) was assayed spectrophotometrically using a commercial kit (CK-NAC BIRG ref. 11.002.00, Biotécnica, Varginha, MG, Brazil). The reactions were run at 37°C and changes in absorbance at 340 nm were monitored with a Shimadzu multispec-1501 spectrophotometer. One unit of activity corresponded to the amount of enzyme that catalyzed 1 *μ*mol of substrate at 25°C.

### 2.7. Statistical Analysis

Each protocol of the pharmacological assays was repeated at least four times and the results are shown as the mean ± SEM. The number of experiments (*n*) is indicated in the figure legends. All statistical comparisons (pharmacological assays and CK determinations) were done using Student's *t*-test with the confidence level set at 5% (*α* = 0.05).

## 3. Results

### 3.1. Betulin Solutions

Table 2 shows the osmolality of PEG 400, the vehicle for betulin dispersion, and betulin solutions. For intravenous injection, 1–3 mg of betulin dispersed in 15–45 *μ*L of PEG 400 would appear to be good combinations since CBA alone had an osmolality of 421 mOsmol/kg. However, since for intravenous injection via a penile vein multiple administrations would be necessary to achieve a total amount of 20 mg of betulin, the intraperitoneal route of administration was selected for use in the* in situ* experiments; this route allowed a single injection of the total amount of betulin required (20 mg in 1 mL).

### 3.2. Contractile Responses in PND Preparations

**Figure 2:**
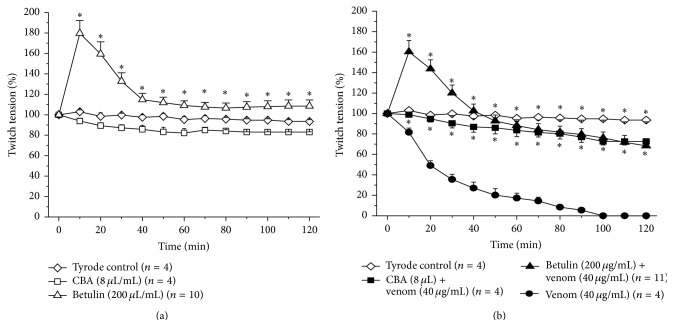
Contractile responses of mouse indirectly stimulated PND preparations incubated with Tyrode solution, CBA, and betulin (a) and* B. jararacussu* venom alone, venom + betulin, and venom + CBA (b). Note the facilitatory effect of betulin (alone and in the presence of venom). The points represent the mean ± SEM of the number of experiments (*n*) indicated in each panel. Note that in several cases the error bars are smaller than the symbol. ^*∗*^ *p* < 0.05 compared to the Tyrode control (a) or venom alone (b). Note that, from 70 min onwards, the asterisks refer to the venom + betulin and venom + CBA curves.

Figure 2 shows the contractile responses of mouse PND preparations incubated with Tyrode solution (*n* = 4), CBA (8 *μ*L/mL, *n* = 4), and betulin (200 *μ*g/mL, *n* = 10) (panel A). Betulin alone caused marked facilitation (80% increase in twitch-tension after 10 min) compared to Tyrode solution and CBA, followed by a progressive decline until 40 min and subsequent stabilization of the tension at a level slightly but significantly above that of saline controls. Panel B shows the response to venom in the absence and presence of CBA and betulin. Venom alone (40 *μ*g/mL) caused progressive, irreversible neuromuscular blockade that was virtually abolished by preincubating the venom with CBA. The preincubation of venom with betulin also markedly attenuated the venom-induced blockade, although it was difficult to compare the responses between the two groups because of the strong facilitation caused by betulin that may have masked the responses to venom (the curves for blockade by venom and venom + CBA were virtually parallel, with the latter simply being displaced in relation to the former because of the betulin-induced facilitation).

### 3.3. Contractile Responses in EPSTA Preparations

**Figure 3:**
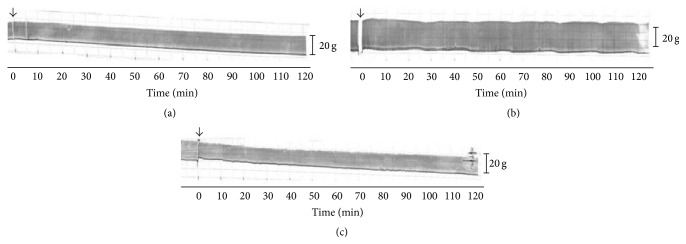
Contractile responses of rat indirectly stimulated EPSTA preparations after treatment with saline (a), CBA (b), and PEG 400 (c). Saline and PEG 400 were injected i.m. in the left hind limb, whereas CBA was injected i.v. The recordings are representative of four experiments for the saline and CBA treatments; only one experiment was done with PEG 400 since we have shown elsewhere that this reagent is compatible with biological preparations [38]. Substances were injected at the arrow. Tension scale bar = 20 g/cm.

The EPSTA preparation has an advantage over the PND preparation in that it is “mounted”* in situ*, with the anesthetized animal being kept alive throughout the experiment. This allows the administration of substances locally (i.m. injection), parenterally (i.p. injection), systemically (i.v. injection), or via a combination of these routes; these possibilities do not exist for PND preparations. However, use of the EPSTA preparation requires rigorous control of all variables, including the solutions to be injected, so as to avoid false positive or negative results.  Figure 3 shows the twitch-tension records for indirectly stimulated EPSTA preparations treated with saline, PEG 400, and CBA, as described in Section 2.5; muscle contractility in the saline-, PEG 400- and CBA-treated preparations was unaltered at the end of the experiments, with twitch-tensions corresponding to 102.7 ± 3.9% (*n* = 4), 98% (*n* = 1; only one experiment was done as screening), and 100 ± 1.6% (*n* = 4) of basal values (time zero), respectively. There were no significant changes in the contractile responses at the end of the incubations with each substance. Thus, any changes seen in subsequent treatments with other agents were not attributable to the effects of saline, PEG 400, or CBA.

**Figure 4:**
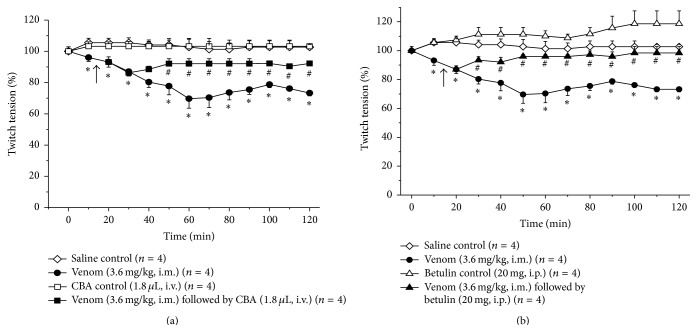
Contractile responses of indirectly stimulated EPSTA preparations treated with saline (i.m.), venom (3.6 mg/kg, i.m., in 120 *μ*L), and venom + CBA (1.8 *μ*L in 1 mL, i.v.) (a) or betulin (20 mg in 1 mL, i.p.) and venom + betulin (b). Note the parallelism between the responses to betulin alone and venom + betulin. The points represent the mean ± SEM of the number of experiments (*n*) indicated in each panel. ^*∗*^ *p* < 0.05 compared to saline and ^#^ *p* < 0.05 compared to venom alone. CBA and betulin were administered at the arrows 15 min after the venom.

Figure 4 shows the contractile responses of EPSTA preparations to venom (3.6 mg/kg, *n* = 4) injected i.m. in the left hind limb. There was progressive blockade from 10 min to 60 min after venom, when the contractile responses stabilized, followed by slight reversal. The maximal blockade at 60 min was 29.5 ± 1.6%. At 120 min, the contractile responses were 73 ± 1.6% of the saline (control) values, that is, significantly below the saline responses. In these protocols, CBA was administered i.v. because this is the route normally used for antivenom injection in the clinical setting and we wished to assess whether CBA given by this route could attenuate the venom-induced neuromuscular blockade. As shown here, CBA administered i.v. 15 min after venom injection significantly attenuated the venom-induced blockade from 50 min onwards. After 120 min, the contractile responses to venom + CBA were 92.2 ± 0.4% of the control responses and significantly greater (*p* < 0.05) than the responses to venom alone (Figure 4(a)). Betulin alone (20 mg in 1 mL of sterile saline) administered i.p. caused progressive muscle facilitation that reached 18.6 ± 8.9% (*n* = 4) at 120 min but was not significantly greater than that seen with saline alone. Betulin injected i.p. 15 min after venom significantly attenuated the venom-induced blockade from 40 min onwards so that, by 120 min after venom, the contractile responses were 98.4 ± 4.4% (*n* = 4) of control (saline) preparations (there was no significant difference between the two treatments at this point). Based on these findings alone, it is unclear whether betulin interacted directly with the venom to neutralize the venom-induced blockade or whether the attenuation of blockade was simply a consequence of the facilitatory effect of betulin on the muscle that masked the venom-induced blockade. However, the finding that the reversal of blockade seen with betulin was similar to that seen with CBA (no significant difference between them), which did not cause facilitation, strengthens the hypothesis of a direct interaction between betulin and venom components.

### 3.4. Myotoxicity Assessed by CK Release

**Figure 5:**
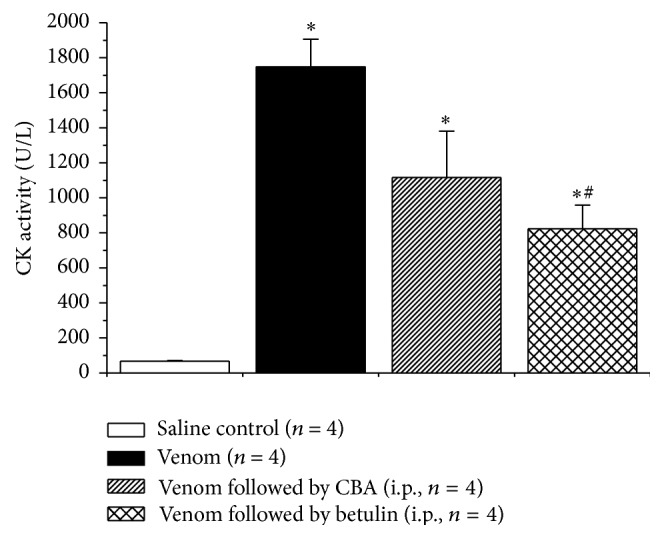
Plasma CK activity 120 min after treatment with saline (negative control, i.m.), venom (positive control, i.m.), venom (i.m.) + CBA (i.p.), and venom (i.m.) + betulin (i.p.). The columns represent the mean ± SEM of the number of experiments (*n*) indicated in the figure. ^*∗*^ *p* < 0.05 compared to saline and ^#^ *p* < 0.05 compared to venom alone.

The myotoxicity of the venom was assessed by quantifying the release of CK (Figure 5). Venom alone significantly increased CK release compared to treatment with saline. This increase was attenuated by CBA and, to a greater extent, by betulin administered 15 min after venom injection. There was no significant difference between the responses to venom + betulin and venom + CBA.

## 4. Discussion

Betulin, an ubiquitous triterpenoid that is found in numerous bushes and trees and that is easily isolated from the bark of birch trees, has more limited medicinal uses than its derivative betulinic acid [29]. To date, betulin has been shown to have anti-inflammatory [29], antiproliferative/antitumor [29, 48], and anti-snake venom [33] activities, the latter against the venoms of* B. jararacussu* and* C. d. terrificus*, two of the medically most important snake species in Brazil.

A major difficulty with betulin and other triterpenes is their poor solubility in polar and nonpolar solvents, which makes the use of solubilizing agents mandatory. Such solvents must be able to solubilize the drug at the desired concentration [49]. For this study, PEG 400 was used to disperse betulin for the experiments* in vitro* and* in situ*. We have previously shown that PEG 400 is compatible with use* in vitro* since a volume of 15 *μ*L in an organ bath volume of 5 mL had no effect on the basal responses of PND preparations [38, 39].

The osmolality of normal serum ranges from 285 to 290 mOsmol/kg (or mOsmol/L) and is maintained by regulating renal water excretion, which in turn is modulated by the antidiuretic hormone vasopressin and development of the sensation of thirst to prevent excessive hypertonicity [50]. The osmolality of peripherally infused solutions was defined by the Infusion Nursing Society as 500 mOsm/L, the upper limit of peripheral vein tolerance, based on a study by Gazitua et al. [51]. However, Payne-James and Khawaja [52] suggested that osmolality should be kept below 1000 mOsmol/kg, although values of up to 1700 mOsmol/kg may be used in parenteral nutrition in conjunction with fine-bore polyurethane catheters [53]. Based on these data and as explained in Section 2.5, a single dose of betulin (20 mg) was used for the experiments* in situ*; since this solution had a higher osmotic pressure (Table 2) than blood, it was administered i.p. rather than i.v.

Ferraz et al. [33] considered betulin to be the best phytochemical among triterpenoids isolated from* D. alata* [15] based on its activity against the neurotoxicity and myotoxicity of* B. jararacussu* and* C. d. terrificus* venoms. As shown here, in preincubation protocols, betulin protected against the neuromuscular blockade caused by* B. jararacussu* venom to an extent similar to that seen with CBA after a 120 min incubation. The protective effect of betulin may be related to its intense facilitatory action that could increase the safety margin of the neuromuscular junction. Indeed, we have generally observed that plant products with a facilitatory action provide the best protection against neuromuscular damage caused by aggressive agents such as snake venoms [33, 54–56].

Antivenom therapy is the recognized standard treatment for envenoming following snakebite but may result in side effects such as anaphylactic shock, pyrogenic reaction, and serum sickness. The inefficacy of antivenoms against the local effects (severe pain, edema, hemorrhage, and necrosis) of envenoming may result in permanent venom-induced scarring and deformity as a consequence of extensive tissue damage [57, 58]. In this context, the efficacy of betulin observed in isolated nerve-muscle preparations [33] and confirmed here (Figure 2(b)) was similar to that of CBA. This finding suggested the need to assess this efficacy in a preparation* in situ*.

The rat EPSTA preparation provides a useful means of evaluating substances in real time since the anesthetized animal is kept alive during the experiment. Interventions in the popliteal nerve culminate in sciatic nerve responses. The popliteal fossa is where the sciatic nerve splits into its two major components, the tibial and common peroneal nerves that travel together within the same nerve sheath [59]. The response of the EPSTA preparation to* B. jararacussu* venom corroborated the paralysis first reported for this venom by Rodrigues-Simioni et al. [34], but at a different level. In mouse PND preparations, the blockade was complete and irreversible whereas, in rat EPSTA preparations, the blockade was partial (~30%) but sustained until the end of the experiment. The results obtained with the EPSTA preparation provide experimental evidence of neuromuscular blockade by* B. jararacussu* venom* in vivo* after local i.m. injection (the most common route of venom inoculation in* Bothrops* bites). The discrepancy between the extent of blockade seen in PND and that seen in EPSTA preparations could potentially reflect differences between the test species (mouse versus rat) and muscles used but is much more likely to be related to (1) the mode of venom application; that is, PND preparations were bathed in a solution of venom so that the whole tissue was in contact with venom whereas in EPSTA the muscle was injected locally with venom so that only part of the preparation was in contact with venom, and (2) the amount of venom used, which was much greater in EPSTA than in PND, for example, 1,224 *μ*g (1.224 mg) injected in the EPSTA muscle of a 340 g rat compared to a total of 200 *μ*g per PND preparation. The relative contribution of each of these factors to the responses observed here remains to be determined. In* Bothrops* snakebites, the local effects are progressive even after venom absorption [60]. This observation suggests that the neuromuscular blockade seen in EPSTA is not purely a myonecrotic event. In agreement with this conclusion, bothropstoxin-I, the main myotoxin from* B. jararacussu* venom, is known to act presynaptically [61].

Comparison of the effects of CBA and betulin indicated that the latter had a faster onset (10 min earlier) against venom-induced neuromuscular blockade than CBA, probably because of the large amount of betulin (20 mg) that was administered as a single injection i.p. compared to CBA which was injected slowly i.v. Overall, however, there was no significant difference between the recovery curves for the two treatments; that is, either treatment was as good as the other.

Myonecrosis at the bite site is an important local effect in* Bothrops* snakebites, primarily through the action of phospholipases *A*_2_ [62] and snake venom metalloproteases (SVMPs) [63]. Histological analysis and CK determination are complementary parameters for assessing venom-induced tissue damage. Since Ferraz et al. [33] have already demonstrated the high potency of betulin against* B. jararacussu* and* C. d. terrificus* venoms using light microscopy, in this study we assessed myotoxicity based only on CK release as an indicator of cell damage [37]. The enzyme activity after 2 h (an interval sufficient for maximal CK release by* B. jararacussu* venom [64]) increased from 66.6 ± 5.0 U/L (saline control, *n* = 4) to 1747 ± 159 U/L (*n* = 4, *p* < 0.05).

When rats were treated with CBA administered i.p., the venom myotoxicity was attenuated to 1116 ± 164 U/L, whereas betulin reduced this myotoxicity to 823 ± 136 U/L, significantly less than venom alone, but still greater than the saline control. Statistical analysis showed the superiority of betulin compared to CBA. An important factor in the lower neutralization of CK release by CBA compared to betulin in these experiments may have been the route (i.p.) of CBA administration. In a comparative study on the influence of the route of CBA administration (i.p. versus i.m.), Agostini Utescher et al. [65] concluded that these two routes were much less efficacious than i.v. injection, in agreement with the official recommendation for use of the latter route in clinical envenomation [66]. Although not directly comparable, the findings with the two protocols tested here also point to better neutralization with i.v. administration since the attenuation of neuromuscular blockade by CBA given i.v. (Figure 4(a)) was better than that of myotoxicity (CK release) by CBA given i.m. (Figure 5); this conclusion generally agrees with i.v. administration being the clinically preferred route. Whereas antivenom neutralizes venom via antigen-antibody reactions, the mechanism by which betulin neutralizes* B. jararacussu* venom is unknown. Of various mechanisms postulated for plant-snake venom interactions, protein precipitation [67] appears unlikely since betulin has no toxic effects [9].

## 5. Conclusion

Based on the results described above, two major conclusions can be drawn. The first and more specific conclusion is that betulin injected i.p. can attenuate the neuromuscular effects of* B. jararacussu* venom by mechanisms that remain to be determined; this neutralizing capacity could be potentially useful for treating* Bothrops* bites in a veterinary setting and possibly also in humans, as a complementary measure to the use of antivenom. The second, more general conclusion is that the EPSTA preparation can be useful for studying the neuromuscular effects of* Bothrops* venoms and their neutralization by plant products. The principal advantage of this preparation over more commonly used nerve-muscle preparations such as the mouse PND is the maintenance of the normal physiological mechanisms of the muscle (because the preparation is* in situ*) that may have a role in modulating the responses to venom, thus providing a better simulation of what occurs following envenomation. This more realistic environment may allow better assessment of the efficacy of potential candidate molecules in neutralizing venom activity.

## Figures and Tables

**Table 1:** Plants containing betulin.

Plant	Reference
*Allophylus longipes*	[6]
*Betula* species	[7–9]
*Caesalpinia decapetala*	[10]
*Calluna vulgaris*	[11]
*Desmodium caudatum*	[12]
*Diospyros cuneata*	[13]
*Dipteryx alata*	[14, 15]
*Dolomiaea souliei*	[16]
*Euphorbia lathyrus*	[17]
*Euphorbia denticulata*	[18]
*Euphorbia lunulata*	[19]
*Ficus foveolata*	[20]
*Garcinia livingstonei*	[21]
*Holoptelea integrifolia*	[22]
*Melodinus hemsleyanus*	[23]
*Osmanthus fragrans*	[24]
*Picriafel terrae*	[25]
*Pseuderanthemum carruthersii *var*. atropurpureum*	[26]
*Pyrus* species	[27]
*Quercus variabilis*	[28]

**Table 2:** Osmolality of injectable betulin solutions.

CBA	Betulin (mg)	PEG 400 (*µ*L)	Sterile saline (final volume: 1 mL)	Osmolality (mOsmol/kg)
1 mL	—	—	—	421
—	—	15	985 *µ*L	343
—	—	45	955 *µ*L	421
—	—	90	910 *µ*L	617
—	—	135	865 *µ*L	802
—	—	225	775 *µ*L	3148
—	—	300	700 *µ*L	3143
—	1	15	~985 *µ*L	323
—	3	45	~955 *µ*L	524
—	6	90	~910 *µ*L	547
—	9	135	~865 *µ*L	939
—	15	225	~775 *µ*L	3120
—	20	300	~700 *µ*L	3092

The osmolality values are the mean of duplicate determinations that varied by ≤10%.
